# The Effect of Crosslinking Degree of Hydrogels on Hydrogel Adhesion

**DOI:** 10.3390/gels8100682

**Published:** 2022-10-21

**Authors:** Zhangkang Li, Cheng Yu, Hitendra Kumar, Xiao He, Qingye Lu, Huiyu Bai, Keekyoung Kim, Jinguang Hu

**Affiliations:** 1 Department of Biomedical Engineering, University of Calgary, 2500 University Dr. NW, Calgary, AB T2N 1N4, Canada; 2 Key Laboratory of Synthetic and Biological Colloids, Ministry of Education, School of Chemical and Material Engineering, Jiangnan University, Wuxi 214122, China; 3 Department of Mechanical and Manufacturing Engineering, University of Calgary, 2500 University Dr. NW, Calgary, AB T2N 1N4, Canada; 4 Department of Chemical and Petroleum Engineering, University of Calgary, 2500 University Dr. NW, Calgary, AB T2N 1N4, Canada

**Keywords:** hydrogel, adhesive strength, crosslinking, hydrogen bonding

## Abstract

The development of adhesive hydrogel materials has brought numerous advances to biomedical engineering. Hydrogel adhesion has drawn much attention in research and applications. In this paper, the study of hydrogel adhesion is no longer limited to the surface of hydrogels. Here, the effect of the internal crosslinking degree of hydrogels prepared by different methods on hydrogel adhesion was explored to find the generality. The results show that with the increase in crosslinking degree, the hydrogel adhesion decreased significantly due to the limitation of segment mobility. Moreover, two simple strategies to improve hydrogel adhesion generated by hydrogen bonding were proposed. One was to keep the functional groups used for hydrogel adhesion and the other was to enhance the flexibility of polymer chains that make up hydrogels. We hope this study can provide another approach for improving the hydrogel adhesion generated by hydrogen bonding.

## 1. Introduction

In several day-to-day applications, adhering materials to each other requires the use of a glue [1]. In this case, these two materials are called adherends, and the glue is adhesive [2]. Hydrogels are highly porous three-dimensional crosslinked polymer networks consisting of hydrophilic polymers that enable hydrogels to retain large amounts of water [2]. However, the use of a glue is not feasible when an attachment between human tissues and hydrogels is desired. In the case of hydrogel adhesion, hydrogels are used as the adherend and adhesive simultaneously [1]. Therefore, an adhesive hydrogel surface should be designed if the hydrogels are used in biomedicine, such as for wound dressing and wearable devices [3,4]. Hydrogel adhesion is central to many applications in medicine and engineering [5,6]; therefore, an ideal hydrogel with excellent adhesive performance has drawn considerable attention [7]. Hydrogel adhesion usually results from surface energy, intermolecular interactions, and near-surface effects [1,2]. Academic researchers have typically focused on the surface of hydrogels or the interface between hydrogels and substrates to create strong hydrogel adhesion [3,8]. The internal structure of hydrogel can also affect the adhesion of the hydrogel’s surface, but a few research studies have explored the relationship between the internal structure of hydrogels and surface adhesion [9,10].

A polymer precursor solution typically consists of different polymer chains. Once these polymer chains connect by crosslinking, the precursor solution will transform into solid hydrogels [11,12]. The crosslinking degree of hydrogels can affect the internal structure of hydrogels, which further results in the change in the mechanical and swelling properties of hydrogels. Usually, a higher crosslinking degree of hydrogels corresponds to a higher compressive and tensile strength and lower equilibrium swelling rate [13,14]. In addition to the mechanical and swelling properties, hydrogel adhesion is one of the key hydrogel properties. It has been widely used in medicine and engineering in recent years [15,16]. Although the hydrogel adhesion inherently relies on engineering the contact surface at soft and hydrated interfaces, we found the adhesion is also affected by the crosslinking degree of hydrogels. Usually, hydrogels can be prepared by different crosslinking methods, mainly including the freezing–thawing cycle, photo-crosslinking and thermal-crosslinking [17,18]. To prove the generality of this finding, polyvinyl alcohol (PVA), polyacrylamide (PAM) and polyvinyl alcohol-bearing styrylpyridinium group (PVA-SbQ) hydrogels, prepared by the freezing–thawing (F-T) cycle, thermal-crosslinking and photo-crosslinking, respectively, were chosen to study the effect of crosslinking on hydrogel adhesion produced by hydrogen bonding in this study. Furthermore, the reason why crosslinking degree could change the hydrogel adhesion is also discussed. We hope this study can provide insights into improving the hydrogel adhesion generated by hydrogen bonding.

## 2. Results and Discussion

### 2.1. The Effect of The Crosslinking Degree of PVA Hydrogels on Hydrogel Adhesion, and Mechanical and Swelling Properties

**Figure 1:**
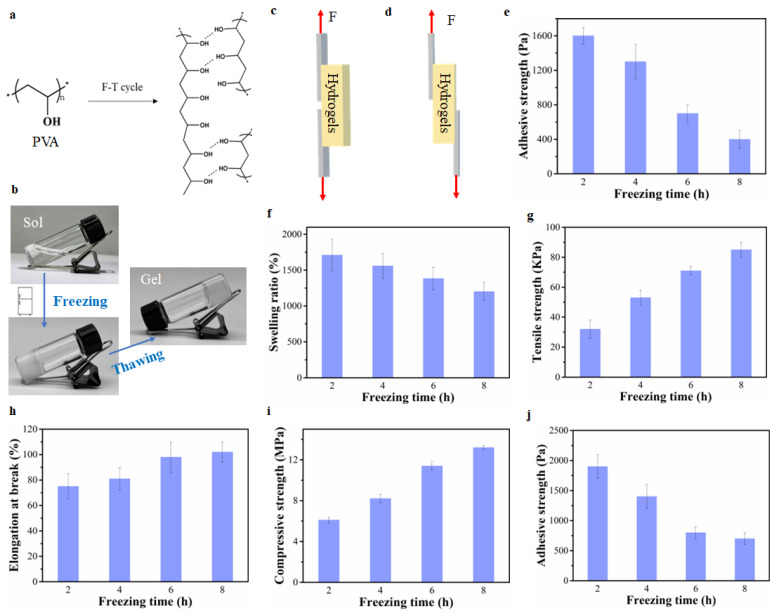
(**a**) The formation mechanism of PVA hydrogels. (**b**) The preparation process of PVA hydrogels. (**c**) The modified method of testing hydrogel adhesion used in this study. (**d**) The general method of testing hydrogel adhesion. (**e**) The adhesive strength of PVA hydrogels prepared by different freezing times. (**f**) The swelling ratio of PVA hydrogels prepared by different freezing times. (**g**) The tensile strength of PVA hydrogels prepared by different freezing times. (**h**) The elongation at break of PVA hydrogels prepared by different freezing times. (**i**) The compressive strength of PVA hydrogels prepared by different freezing times. (**j**) The adhesive strength of PVA/CNC hydrogels prepared by different freezing times.

PVA is a conventional raw material for preparing hydrogels because of its satisfactory biocompatibility, biodegradation, and nontoxicity [19,20]. PVA hydrogels can be formed through the well-known F-T method via the physical crosslinking by hydrogen bonds (Figure 1a,b) [21,22]. During the freezing process, water freezes and reduces interaction with hydroxyl groups on PVA chains [23]. As a result, the hydroxyl groups on PVA chains bind to form hydrogen bonding (Figure 1a). Consequently, the PVA solution becomes a hydrogel due to the development of a 3D structure (Figure 1b). With the increased freezing time, there is an increase in the hydrogen bond formation. Hydrogen bonds act as physical crosslinking points in PVA hydrogels. Therefore, the crosslinking degree depends on the freezing time.

There are several methods of testing hydrogel adhesion [1]. Figure 1c shows the modified method for testing the shear adhesive strength of PVA hydrogels used in this study unlike the general method (Figure 1d) [1,24]. The adherends were stuck to the same side of hydrogels to avoid significant errors due to the low adhesive strength of PVA hydrogels. Figure 1e shows that with the increase in the freezing time of PVA hydrogels, the adhesive strength decreased from 1600 Pa to 400 Pa. In addition, the swelling ratio of PVA hydrogels decreased with the increase in crosslinking degree that results from the increased freezing time (Figure 1f). Conversely, the tensile strength, elongation at break and compressive strength of PVA hydrogels improved with longer freezing duration because of the increased crosslinking degree (Figure 1g–i). It is well-known that the crosslinking degree benefits mechanical strength because a higher crosslinking degree results in a denser hydrogel structure [12,25,26]. In addition to mechanical strength, the crosslinking degree affects hydrogel adhesion simultaneously. For PVA hydrogels prepared by the F-T cycle, on the one hand, most of the hydroxyl groups on PVA formed hydrogen bonds used for crosslinking during the F-T process, which resulted in the lack of free hydroxyl groups used for adhesion [7,27]. On the other hand, the increase in the crosslinking degree of the PVA hydrogels limited the accessibility of hydroxyl groups due to the decrease in segment mobility. Consequently, the adhesive property between hydrogels and substrates reduced significantly with increased freezing time. This phenomenon was also applied to composite hydrogels. As shown in Figure 1j, the adhesion of polyvinyl alcohol/cellulose nanocrystal (PVA/CNC) composite hydrogels decreased with the increase in freezing duration.

### 2.2. The Effect of The Crosslinking Degree of PAM Hydrogels on Hydrogel Adhesion, and Mechanical and Swelling Properties

**Figure 2:**
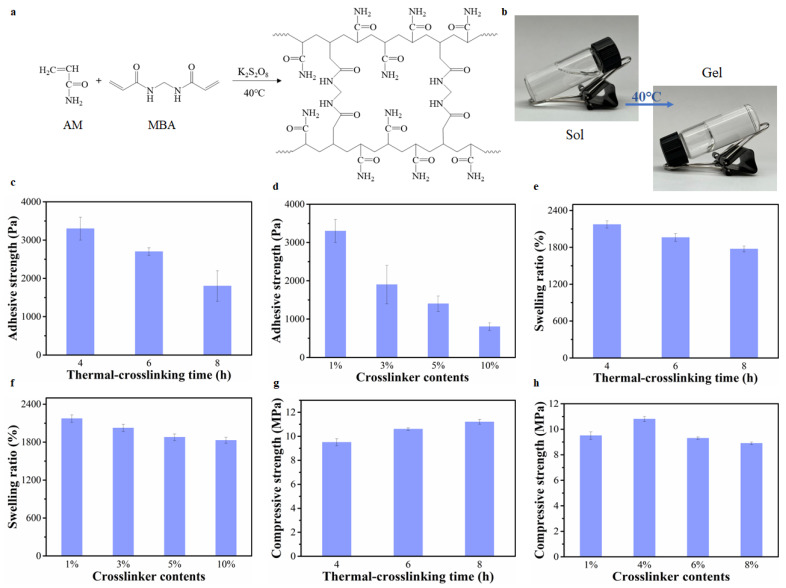
(**a**) The formation mechanism of PAM hydrogels. (**b**) The preparation process of PAM hydrogels. (**c**) The adhesive strength of PAM hydrogels prepared by different thermal-crosslinking times. (**d**) The adhesive strength of PAM hydrogels prepared with different crosslinker contents. (**e**) The swelling ratio of PAM hydrogels prepared by different thermal-crosslinking times. (**f**) The swelling ratio of PAM hydrogels prepared with different crosslinker contents. (**g**) The compressive strength of PAM hydrogels prepared by different thermal-crosslinking times. (**h**) The compressive strength of PAM hydrogels prepared with different crosslinker contents.

Unlike PVA hydrogels prepared by the F-T cycle, PAM hydrogels are usually synthesized by thermal-crosslinking [28,29]. The covalent bonding formed by the polymerization acts as crosslinking points in these types of hydrogels rather than hydrogen bonding (Figure 2a), which results in the sol–gel transition (Figure 2b). The crosslinking degree depends on the crosslinker content and the thermal-crosslinking time. Therefore, PAM hydrogels with different crosslinking degrees were obtained by adjusting crosslinking time and crosslinker content to investigate the relationship between crosslinking degree and hydrogel adhesion. As shown in Figure 2c,d, with the increase in the crosslinking time and crosslinker content, the hydrogel adhesion decreased. This observation implied that a higher crosslinking degree was not helpful for hydrogel adhesion. Unlike the PVA hydrogels, the free functional groups used for adhesion did not decrease in PAM hydrogels [1,2]. The decrease in hydrogel adhesion mainly resulted from the limitation of segment mobility. Although there were a lot of free amino groups on the PAM hydrogels, the amino group could not move to the hydrogel surface to interact with some functional groups on substrates as the crosslinking degree of PAM hydrogels increased. In addition, with the increase in crosslinking time and crosslinker content, the swelling ratio of PAM hydrogels simultaneously decreased (Figure 2e,f). The decreased swelling ratio further corroborated that the crosslinking degree of the hydrogel increased and a denser hydrogel structure was formed [30,31,32]. With the increase in crosslinking degree caused by increased crosslinking time, the compressive strength of PAM hydrogels gradually increased (Figure 2g). Conversely, while the increase in the crosslinker content improved the crosslinking degree, the compressive strength of PAM hydrogels did not increase significantly (Figure 2h). This is because the higher crosslinker content resulted in the crosslinking of PAM without complete polymerization. Accordingly, the length of PAM polymer chains that make up the hydrogel network were short, which resulted in the slight decrease in compressive strength. Therefore, it is challenging to enhance the mechanical and adhesive properties of hydrogels simultaneously.

### 2.3. The Effect of The Crosslinking Degree of PVA-SbQ Hydrogels on Hydrogel Adhesion, and Mechanical and Swelling Properties

**Figure 3:**
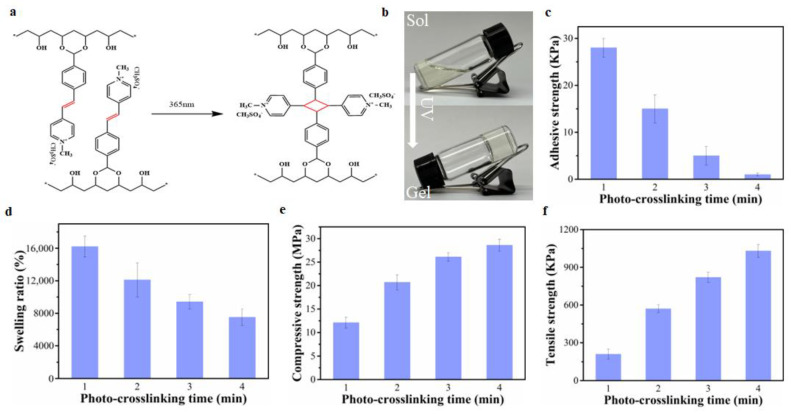
(**a**) The formation mechanism of PVA-SbQ hydrogels. (**b**) The preparation process of PVA-SbQ hydrogels. (**c**) The adhesive strength of PVA-SbQ hydrogels prepared by different photo-crosslinking times. (**d**) The swelling ratio of PVA-SbQ hydrogels prepared by different photo-crosslinking times. (**e**) The compressive strength of PVA-SbQ hydrogels prepared by different photo-crosslinking times. (**f**) The tensile strength of PVA-SbQ hydrogels prepared by different photo-crosslinking times.

In addition to the F-T cycle and thermal-crosslinking, photo-crosslinking is another common method for preparing hydrogels. To prove the generality of the effect of crosslinking degree on hydrogel adhesion, the PVA-SbQ hydrogels with different degrees were prepared by adjusting the photo-crosslinking duration. Instead of the conventional F-T cycle or using chemical crosslinkers, PVA-SbQ hydrogels could be formed by a fast and facile photo-crosslinking method via the photodimerization of carbon–carbon double bonds (C=C bonds) on SbQ functional groups (Figure 3a,b) [33,34]. As shown in Figure 3c, the adhesion of a PVA-SbQ hydrogel sharply decreased with the increase in photo-crosslinking time. The increased photo-crosslinking time also reduced the swelling ratio of PVA-SbQ hydrogels, which suggested an increase in the crosslinking degree (Figure 3d) [30,31,32]. Conversely, the compressive strength and tensile strength steadily increased as the photo-crosslinking duration was increased (Figure 3e,f). As with the PAM hydrogels, the decrease in hydrogel adhesion primarily resulted from the limitation of segment mobility, and the enhanced mechanical properties were caused by the increase in the crosslinking degree. This proved that photo-crosslinked hydrogels also followed the aforementioned relationship between the degree of crosslinking and hydrogel adhesiveness.

### 2.4. The Strategies for Constructing Strong Hydrogel Adhesion

**Figure 4:**
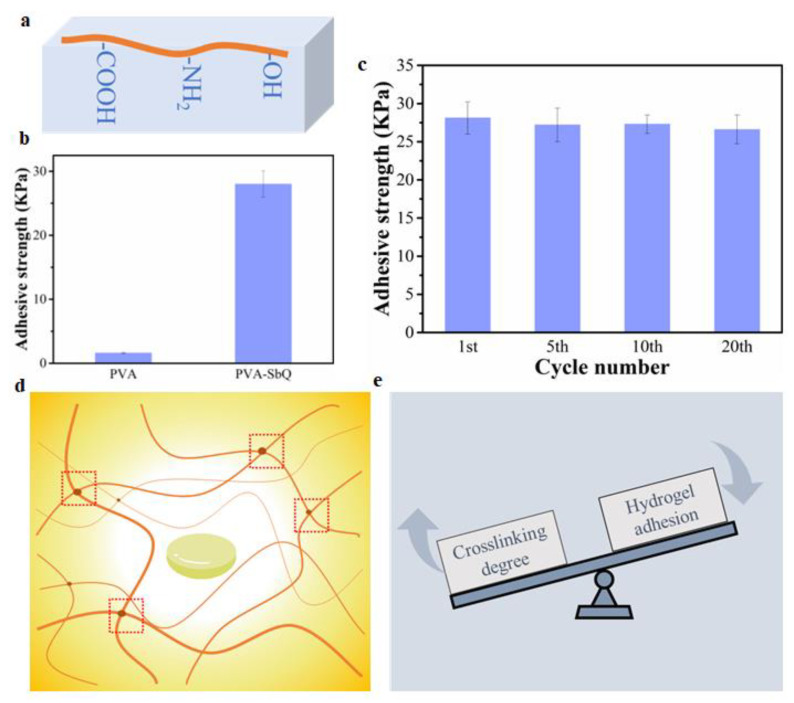
(**a**) The functional groups required for hydrogel adhesion generated by hydrogen bonds. (**b**) The maximum adhesive strength of PVA and PVA-SbQ hydrogels. (**c**) The adhesive strength of PVA-SbQ hydrogels under different stripping cycles. (**d**) The effect of crosslinking degree of hydrogels on chain movement. (**e**) The effect of crosslinking degree on hydrogel adhesion.

In the study, three kinds of hydrogels were prepared by the freezing–thawing cycle, thermal-crosslinking and photo-crosslinking, respectively. The hydrogel adhesion was primarily generated by hydrogen bonding because of the amino and hydroxyl groups. In addition to the amino and hydroxyl groups shown in these hydrogels, carboxyl groups can also contribute towards hydrogel adhesion. Therefore, amino, hydroxyl or carboxyl groups are required to construct hydrogel adhesion generated by hydrogen bonding (Figure 4a). However, these functional groups should be free. For instance, the maximum adhesive strength of PVA-SbQ hydrogels is much higher than that of PVA hydrogels (Figure 4b). Although there were many hydroxyl groups in PVA hydrogels, they formed hydrogen bonding used for crosslinking with themselves rather than interacting with the functional groups on the substrate. As a result, these hydroxyl groups are not free and will contribute towards hydrogel adhesion. On the contrary, the fabricated PVA-SbQ hydrogel holds massive free hydroxyl groups as the crosslinking was mainly driven by photodimerization of SbQ groups instead of hydrogen bonds forming between hydroxyl groups. The advantage of hydrogen bond-triggered adhesion is that it is recyclable since the hydrogen bonding formation utilizes dynamic and noncovalent interactions. The cyclic stripping test demonstrated that the adhesive strength of the PVA-SbQ hydrogel did not reduce significantly with the increase in the number of stripping cycles (Figure 4c). In addition to the free functional groups, it was found that the crosslinking degree also affected the hydrogel adhesion. The increase in the crosslinking degree of the hydrogel restricted the mobility of the polymer chains (Figure 4d). Hence, the polymer chains with functional groups were not quickly diffused towards the hydrogel surface to form intimate contact with the substrate. As a result, excessive crosslinking of hydrogels led to a reduced adhesive strength (Figure 4e).

## 3. Conclusions

In this study, we investigated PVA, PAM and PVA-SbQ hydrogels fabricated by the freezing–thawing cycle, thermal-crosslinking and photo-crosslinking, respectively. These three types of hydrogels present representative models of hydrogels formed by different crosslinking mechanisms and extend the observations and derived conclusion to a wide class of materials. The hydrogel adhesion capability decreased with the increase in the crosslinking degree of these hydrogels. This is because a higher crosslinking degree would limit segment mobility. As a result, functional groups on polymer chains could not move to the hydrogel surface to interact with the substrate to generate hydrogel adhesion. Therefore, in addition to free functional groups used to interact with substrates, the flexibility of polymer chains that make up hydrogels is vital for hydrogel adhesion. However, the decrease in crosslinking degree in order to increase the availability of free functional groups and higher segment mobility would result in poor mechanical strength of the hydrogels and limit their functionality. Therefore, it is essential as well as challenging to maintain the balance between hydrogel adhesion and mechanical strength. However, a rational design of a hydrogel crosslinked network and free functional groups can allow better control over these hydrogel characteristics.

## 4. Materials and Methods

### 4.1. Materials

Polyvinyl alcohol (PVA, Mn ≈ 130,000, 99% hydrolysis), N,N’-methylenebisacrylamide (MBA), potassium persulfate (KPS) and acrylamide (AM) were obtained from Sigma-Aldrich (St. Louis, MO, USA) and used without further purification. Polyvinyl alcohol-bearing styrylpyridinium group (PVA-SbQ) solution was purchased from Shanghai KCKI Printing Technology Co., Ltd., Shanghai, China (polymerization degree was 1700, and the concentration of SbQ was 0.03–0.05 mol kg^−1^), and cellulose nanocrystal (CNC) aqueous suspension was prepared from cotton linters.

### 4.2. The Preparation of PVA Hydrogel

First, 8.0 g PVA powder was added to 92 g deionized water to obtain 8 wt% PVA solution. Secondly, the mixtures were put into in a three-mouth flask and then stirred at 95 °C until the PVA powder was completely dissolved. Subsequently, the PVA solution was cooled down to room temperature, until the air bubbles completely disappeared. Lastly, 8 wt% PVA solution was poured into a watch glass and placed in a −20 °C refrigerator for 2, 4, 6 and 8 h respectively to obtain PVA hydrogels with different freezing durations.

### 4.3. The Preparation of PVA/CNC Hydrogels

First, the CNC aqueous suspension was prepared by acid hydrolysis method. Specific steps are as follows: 3 g cotton fiber was added to 62 mL 65% H_2_SO_4_ and treated at 50 °C for 45 min under constant stirring. The mixture was centrifuged and dialyzed against deionized water to remove the residual acid. The aqueous suspension was then ultrasonicated to evenly disperse the CNC and break agglomerates. The dispersed CNC was diluted to obtain a homogenous aqueous dispersion.

Next, 8 wt% PVA solution was added to the CNC aqueous suspension at given CNC/PVA mass ratio of 0.01:1. Subsequently, the PVA/CNC solution was stirred at 90 °C for 2 h and then ultrasonicated for additional 20 min. Finally, the PVA/CNC solution was poured into a watch glass and placed in a −20 °C refrigerator for 2, 4, 6 and 8 h, respectively, to obtain PVA/CNC hydrogels with different freezing durations.

### 4.4. The Preparation of PAM Hydrogels

An 8 wt% AM solution was obtained by dissolving 8 g AM in 92 g deionized water. Subsequently, different weights of MBA powders were added into the 8 wt% AM solution to obtain samples with different compositions, where the mass ratio between MBA and AM were 1:100, 3:100, 5:100 and 10:100, respectively. These mixtures were then stirred continuously for 30 min. After the addition of the MBA and stirring for 30 min, 0.0192 g KPS was added into the solution and stirred for another 10 min to initiate polymerization. Finally, the prepared hydrogel solutions were placed at a temperature of 45 °C for 4 h to obtain PAM hydrogels with different MBA compositions. Among the various hydrogels, the PAM hydrogels with 1% MBA were selected to be placed in 45 °C for 4, 6 and 8 h, respectively, to obtain and study PAM hydrogels with different thermal-crosslinking times.

### 4.5. The Preparation of PVA-SbQ Hydrogels

PVA-SbQ hydrogels were directly formed from 8 wt% PVA-SbQ solution with different photo-crosslinking times by being exposed to an F300 UV lamp for 1, 2, 3, 4 min, respectively.

### 4.6. Characterization

Tensile strength of the hydrogels was evaluated by performing tensile tests on an Instron 5967 electronic universal testing machine. The PVA and PVA/SbQ hydrogels were cut into dumbbell-shaped samples with a diameter of 7–9 mm and a thickness of 3–5 mm. Additionally, the compressive tests were carried out on cylindrical hydrogel samples with a diameter of 7–9 mm and a thickness of 3–5 mm by an Instron 5967 electronic universal testing machine at room temperature. The compression rate was fixed at 2 mm/min. Measurements of each sample were repeated at least three times and the acquired data were reported as average value and standard deviation.

To estimate the adhesive strength of hydrogels, the shear adhesive strength tests were performed. The PVA, PVA/CNC, PAM and PVA/SbQ hydrogels were cut into a rectangular spline with a size of 40 mm × 10 mm × 5 mm and then adhered to metal sheets, where the adhesion area on one side was 10 mm × 10 mm and the other side was 10 mm × 20 mm, as shown in Figure 1c. The force response was recorded while keeping one metal sheet fixed and moving another metal sheet at a speed of 20 mm/min using a universal testing machine. In the experiment, 5 samples were tested in each group to obtain the average value and standard deviation. The adhesive strength was calculated by the maximum load divided by the adhesion area.

To test the swelling ratio of hydrogels, the hydrogel samples were immersed in distilled water for 3 days at least at room temperature, until the hydrogel weight did not increase. In the next step, these swollen hydrogels were placed in a 40 °C oven to evaporate all of the water in hydrogels. The weight of swollen hydrogel and dried hydrogels were recorded, respectively. The equilibrium swelling ratio was calculated by the following equation

ESR% = W_s_/W_d_ × 100 (1)

where W_s_ and W_d_ are the weight of the swollen hydrogel at room temperature and dried hydrogels, respectively.

## Data Availability

Not applicable.
